# Patient-important outcomes in systematic reviews: Poor quality of evidence

**DOI:** 10.1371/journal.pone.0195460

**Published:** 2018-04-05

**Authors:** Youri Yordanov, Agnes Dechartres, Philippe Ravaud

**Affiliations:** 1 INSERM, U1153, Paris, France; 2 Sorbonne Universités, UPMC Paris Univ-06, Paris, France; 3 Service des Urgences - Hôpital Saint Antoine, Assistance Publique–Hôpitaux de Paris (APHP), Paris, France; 4 Centre d’Épidémiologie Clinique, Hôpital Hôtel Dieu, Assistance Publique–Hôpitaux de Paris (APHP), Paris, France; 5 Faculté de Médecine, Université Paris Descartes, Sorbonne Paris Cité, Paris, France; 6 Cochrane France, Paris, France; 7 Columbia University, Mailman School of Public Health, Department of Epidemiology, New York, United States of America; Universita degli Studi di Firenze, ITALY

## Abstract

**Background:**

Cochrane reviewers are strongly encouraged to evaluate the quality of evidence for the most important outcomes by using the GRADE approach and to report these results in a Summary of Findings (SoF) table. We aimed to assess whether outcomes reported in the SoF table of Cochrane reviews could be considered patient-important outcomes (PIOs) and the quality of the available evidence for these outcomes.

**Methods:**

We performed a methodological review of Cochrane reviews published between March 2011 and September 2014. For a random sample of Cochrane reviews reporting a SoF table, we extracted all outcomes reported in this table and evaluated whether they could be considered PIOs (i.e., mortality, other clinical events, adverse events, function, pain, quality of life and therapeutic decisions). Then, we collected the quality of evidence for every outcome in these SoF tables.

**Results:**

We included 290 reviews issued by 47 of the 53 Cochrane Review Groups. Every SoF table included a median of 5 outcomes, for a total of 1414 outcomes; 1089 (77%) could be considered PIOs. Almost all reviews (n = 278, 96%) included at least one PIO in their SoF table. The quality of evidence for the outcomes was high for 12% (n = 168), moderate for 28% (n = 402) and low or very low for 45% (n = 640). Less than one quarter of reviews (n = 63) included at least one PIO with high-quality evidence that favoured a benefit of the experimental intervention evaluated in half of them (n = 34 reviews).

**Conclusions:**

Many outcomes reported in the SoF table of recent Cochrane reviews can be considered PIOs. However, the quality of available evidence remains limited for these outcomes.

## Introduction

In the last decade, clinicians and researchers have been encouraged to recommend healthcare interventions based on their effect on patient-important outcomes (PIOs) such as death, other clinical events, quality of life or functional outcomes[1–6]. However, several methodological reviews reported that the use of PIOs in randomized controlled trials (RCTs) of various medical specialities is still far from optimal [7–10]. In a recent report assessing trials of critically ill patients, less than one quarter of primary outcomes (24%) were PIOs[9]. This proportion was as low as 5% when exploring outcomes besides mortality after intensive care unit discharge, such as functional disability or quality of life[9]. Similar results were previously found for diabetes[8] or cardiovascular [7] trials, with 18% and 23% of these trials assessing PIOs. In contrast, some recent studies suggested that systematic reviews are more likely to evaluate PIOs than their individual trials[10, 11].

A key component of systematic reviews is the elaboration of a clear and focused clinical question by specifying the types of participants, interventions, comparisons, and outcomes that should be considered[12, 13]. When planning a Cochrane review, authors are strongly encouraged to consider all outcomes that are meaningful to patients, physicians, policy makers or any other health care stakeholders, regardless of their availability in individual trials [13, 14]. When reporting the review, they are asked to present a Summary of Findings (SoF) table summarizing the quality of evidence and treatment effect magnitude for the most essential outcomes for decision-making by using the Grading of Recommendations Assessment, Development and Evaluation (GRADE) approach [13–18].

In this study, we aimed to evaluate: 1) whether outcomes reported in the SoF table could be considered PIOs and 2) the quality of evidence for these outcomes in a sample of recent Cochrane reviews.

## Methods

This is a methodological review of recently published Cochrane reviews. For each Cochrane review reporting an SoF table, we extracted all outcomes reported in this table and evaluated whether they could be considered PIOs. Then, we evaluated the quality of evidence for these outcomes.

### Data sources

We previously obtained data from all systematic reviews published between March 2011 and September 2014 from the Cochrane Collaboration [19]. Data were provided as XML files and contained all information reported by the review authors using RevMan[20], the review software developed by the Cochrane Collaboration to prepare and update systematic reviews.

### Review selection

Using R 3.2.2 and the XML package, we identified all reviews of RCTs including at least one SoF table. Reviews including observational studies were excluded. We also excluded reviews including only trials published before 2007 to focus on recent topics. Then, we manually examined all review titles and abstracts when necessary to select those evaluating a healthcare intervention (pharmacological or non-pharmacological). Reviews evaluating diagnostic test accuracy or economic evaluations were excluded. Among the set of eligible reviews, by using a random number generator, we drew a random sample of 300 reviews for comprehensive evaluation.

### Identification of the SoF for the main comparison

Most reviews have a single SoF table, but some have several, corresponding to different comparisons. In this case, we manually identified the main comparison as reported by the review authors. If no main comparison was identified as such, we selected the one with the largest number of available outcomes and included the largest number of trials. When the same SoF table reported various comparisons, the review was excluded.

### Data collection

For every included systematic review, we collected the following characteristics by using a standardized data extraction sheet:

- Review general characteristics: title, digital object identifier (DOI), protocol publication date, review first publication date, Cochrane Review Group (e.g., Gynaecological Cancer Group), type of intervention (i.e., pharmacological or non-pharmacological), number of excluded and included trials.
- Characteristics of the main comparison: Patient or population description, setting, intervention and comparator
- Every outcome reported in the SoF table including outcome description and follow-up period.

### Classification of outcomes

We categorized each outcome as follows: mortality, other clinical events (e.g., myocardial infarction or stroke), therapeutic decision (e.g., transfusion), function (e.g., anxiety, depression, disability and dyspnoea), pain, quality of life, adverse events or side effects (identified as such by the review authors), physiological parameters (e.g., blood pressure, weight), biological parameters (e.g., cholesterol levels), radiological parameters (e.g., measure of joint space), compliance (e.g., discontinuation for any reason), process (e.g., duration of surgical procedure), resource use (hospitalisations), cost-effectiveness and satisfaction with care. A single reviewer classified all outcomes. As a quality measure, 10% of the outcomes were classified independently in duplicate (YY and AD).

### Definition of PIOs

We considered mortality, other clinical events, adverse events, function, pain, quality of life and therapeutic decisions as PIOs consistent with previous studies [8, 11, 21, 22].

### Quality of evidence

We evaluated the quality of evidence of each outcome reported in the SoF table based on the review authors assessment. The evidence was classified as high quality if further research is very unlikely to change the authors confidence in the estimate of effect; moderate quality if further research is likely to have an important impact on the authors’ confidence in the estimate of effect and may change the estimate; low quality if further research is very likely to have an important impact on the confidence in the estimate of effect and likely to change the estimate; and very low quality if the estimate of the effect is very uncertain[18]. We evaluated separately the quality of evidence for outcomes that could be considered PIOs. For those considered with high quality of evidence, we also evaluated whether this was evidence of benefit for the experimental intervention (results in favour of the experimental intervention: i.e., statistically significant results).

### Statistical analysis

The analysis was mostly descriptive. Continuous data are presented as median (Q1–Q3) and qualitative data as frequency (percentage). All analyses involved use of R v3.1.1 (R Foundation for Statistical Computing, Vienna, Austria. http://www.R-project.org/).

## Results

### Selection of relevant Cochrane systematic reviews

The selection process was previously described[19]. Briefly, between March 2011 and September 2014, 2796 Cochrane systematic reviews were published, 1670 did not include an SoF table (60% of screened reviews) and 820 were eligible for inclusion. We identified a random sample of 300 reviews, but 10 were additionally excluded because their SoF presented different interventions within the same table, which left 290 reviews for further evaluation.

### Characteristics of included reviews

The reviews were issued by 47 of the 53 Cochrane Review Groups[23]. Every review included a median of 11 trials overall (Q1-Q3: 5–21), with a median of 5 trials per main comparison (Q1-Q3: 3–12)[19]. The experimental intervention was non-pharmacological in 40% of the reviews (n = 115). Every SoF table included a median of 5 outcomes (Q1-Q3: 3–7), for a total of 1414 outcomes. The corresponding meta-analyses included a median of 3 trials (Q1-Q3: 2–7).

### Outcome classification

**Fig 1:**
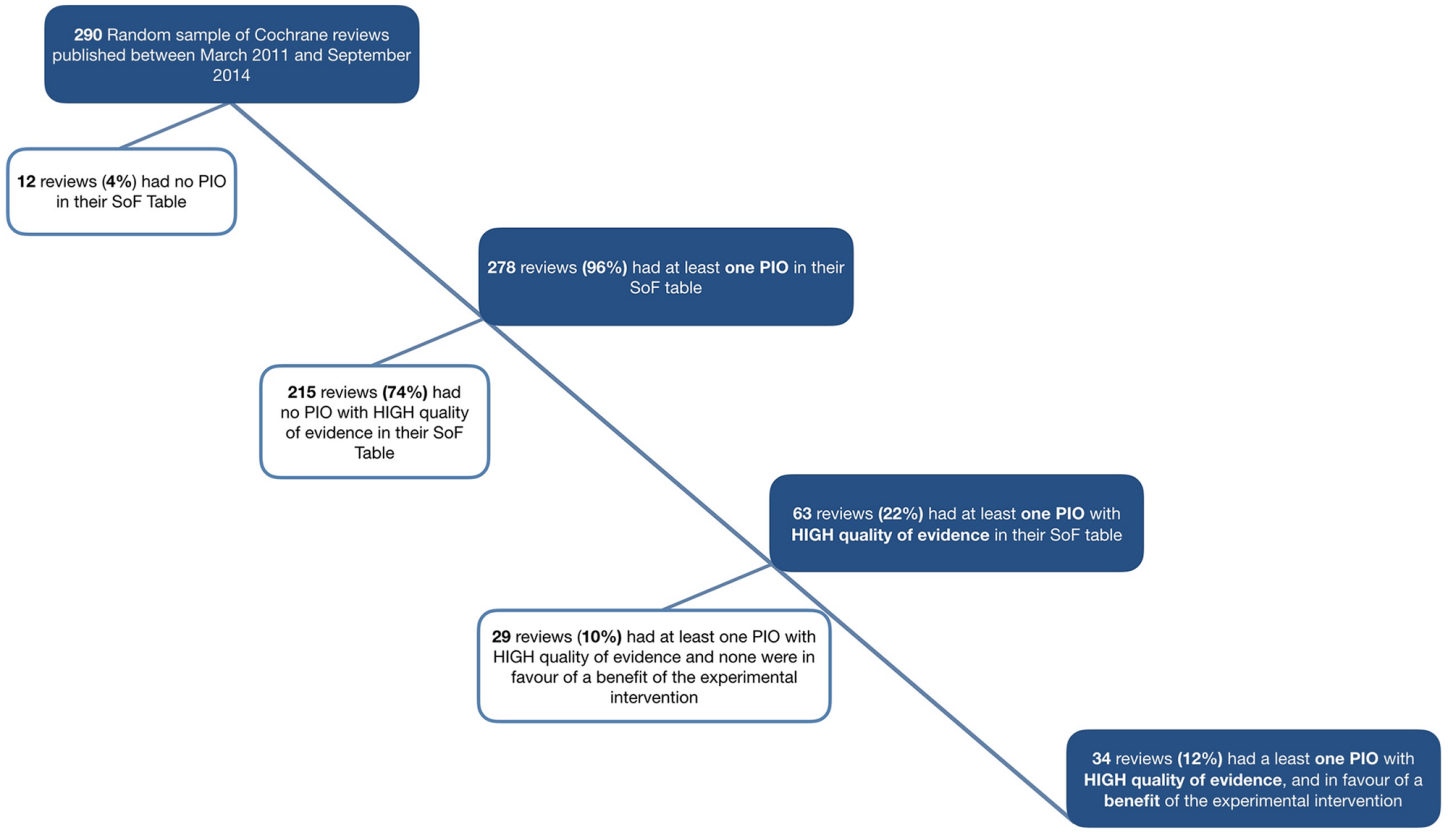
Patient-important outcomes in a random sample of Cochrane reviews.

Among the 1414 outcomes reported in the SoFs, the most common were functional outcomes (27%, n = 384), clinical events (14%, n = 198) and adverse events (12%, n = 174). Mortality represented 10% (n = 138) of the outcomes and quality of life 7% (n = 98). Among the outcomes, biological parameters represented 6% (n = 89), process and resource use 5% (n = 74), and physiological parameters and compliance 4% (n = 56) and 3% (n = 45). A total of 1089 outcomes (77%, 95% CI: 75–79) could be considered PIOs (Table 1). Almost all reviews included at least one PIO in the SoF table (n = 278, 96%; 95% CI: 93–98) (Fig 1). Per review, the median proportion of PIOs among the outcomes reported in the SoF was 86% (Q1-Q3: 67–100).

**Table 1:** Classification of outcomes reported in the Summary of Findings (SoF) table in 290 recent Cochrane reviews.

	No. (%)
	N = 1414 *
**Patient-important outcomes (PIOs)**	
Function	384 (27%)
Other clinical events	198 (14%)
Adverse events—side effects	174 (12%)
Mortality	138 (10%)
Quality of life	98 (7%)
Pain	71 (5%)
Therapeutic decision	33 (2%)
PIOs among the SoF table outcomes	1089 (77%; 95% CI 75–79)
**Other outcomes**	
Biological parameter	89 (6%)
Process, resource use	74 (5%)
Physiological parameters	56 (4%)
Compliance	45 (3%)
Satisfaction with care	24 (2%)
Radiological parameters	23 (2%)
Cost-effectiveness	16 (1%)

*The total is higher than N = 1414 because some outcomes were included in more than one category 95% CI, 95% confidence interval

### Quality of available evidence

The quality of evidence provided by the outcomes in the SoF tables was high for 12% (n = 168, 95% CI: 10–14) and moderate for 28% (n = 402, 95% CI: 26–31) (Table 2). For 45% of the outcomes (n = 640, 95% CI: 43–48), the quality of the evidence was low or very low. No GRADE assessment was available for 12% of the outcomes (n = 164, 95% CI: 10–13) because according to the review authors, these outcomes were not reported or measured in any individual trials. The quality of the available evidence was very close when focusing on only PIOs, with 41% (n = 449) having high and moderate evidence and 44% (n = 475) low to very low evidence.

**Table 2:** Quality of available evidence for outcomes reported in the SoF tables for the main comparison in 290 recent Cochrane reviews.

		All outcomes	PIOs
		N = 1414	N = 1089
Quality of evidence	Proportion in %	Proportion in %	Proportion in %
High quality	Further research is very unlikely to change our confidence in the estimate of effect.	12	12
Moderate quality	Further research is likely to have an important impact on our confidence in the estimate of effect and may change the estimate.	28	29
Low quality	Further research is very likely to have an important impact on our confidence in the estimate of effect and likely to change the estimate.	28	27
Very low quality	We are very uncertain about the estimate.	17	16
No GRADE	No individual trial reported or measured the desired outcome	12	12
No GRADE	Other reasons	3	3

Less than one-quarter of the reviews, 22% (n = 63; 95% CI: 17–27), included at least one PIO with evidence considered of high quality by the review authors. In half (n = 34) of these reviews, the results were in favour of the experimental intervention (i.e., statistically significant results) (Fig 1).

## Discussion

In this study, we evaluated whether outcomes reported in the SoF table of recent Cochrane reviews could be considered PIOs and the quality of evidence provided for these outcomes. The large variety of review groups represented allowed us to explore very different medical specialties. More than three-quarters of the outcomes reported in the SoF tables could be considered PIOs. However, for a large proportion of the available evidence (45%), the quality was considered low or very low. About one quarter of the reviews included at least one PIO with high-quality evidence and for half of them, this evidence showed a benefit of the experimental intervention.

The Cochrane Collaboration strongly encourages review authors to include SoF tables to present their main findings [13]. These tables, first introduced in Cochrane reviews in 2008, aim to synthesize in a simple, transparent and accessible format, key information on the assessed interventions’ magnitude of effect, sum of available data for the main outcomes and quality of evidence [13]. Therefore, SoF tables should include the most important outcomes whether they were measured in individual trials or not[13]. Previous studies reported that as compared with reviews without SoF tables, the inclusion of SoF tables seemed to improve readers’ general understanding of the reviews and allowed them to better identify the critical information and find results for important outcomes (93% vs 44%)[24]. However, not all Cochrane reviews present a SoF table. In our study, we excluded 60% of Cochrane reviews because they had no SoF table, which is consistent with a previous study showing a proportion of inclusion of SoF tables evolving from 31% in 2008 to 57% in 2013 [25]. An alternative version of the SoF table called the interactive SoF (iSoF) allowing review authors to choose alternative displays (absolute effects rather than relative effects) is being tested[26], which may help generalise their use.

We found that most of the outcomes reported in the SoF tables could be considered PIOs, with a median proportion of PIOs in the SoF tables of 86%. In a study that focused on recently published Cochrane and non-Cochrane reviews but also on registered systematic reviews protocols (PROSPERO), Ameur *et al* described that 95% of Cochrane reviews included at least one PIO among their primary outcomes. This proportion was higher than for non-Cochrane reviews[11]. Two previous studies also found consistent results, with 68% to 71% of the reviews considering PIOs as primary outcomes[21, 22]. A large survey of the completeness of main outcomes mapped the availability of information for a major clinical outcome across randomized trials and systematic reviews in an entire discipline: 75% of reviews reported the major clinical outcome as compared with only 20% of primary trial reports [27]. All these results highlight the gap in outcomes between systematic reviews and their individual trials, which may explain why the quality of evidence was frequently limited for the outcomes reported in the SoF table, as shown by our results. In our study, only 22% of the reviews had at least one PIO with a high level of evidence, with half showing a benefit of the experimental intervention. The failure to consider PIOs in clinical trials may lead to erroneous evaluation of benefits, with possible serious consequences for patients, and represents waste[28–31]. To improve this situation, core outcome sets (COSs) should be developed (ie, standardized sets of outcomes, that have been agreed upon, and that should be measured and reported in every trial for a specific healthcare condition) and their use promoted [32–34]. The number of COSs developed is still limited but is progressively increasing, such as their use in systematic reviews [11, 25, 33, 35–37].

Our study has limitations. Although we relied on detailed and previously published definitions for PIOs, considerable judgment remains when assessing the importance of the outcomes, and patients or experts in the assessed medical field were not involved in this evaluation. Our results cannot be generalized to all systematic reviews because Cochrane reviews have precise conduct and reporting guidelines[13–15] and were described as more transparently reported than all other types of systematic reviews[38]. Our study confirms that many outcomes reported in the SoF tables of recent Cochrane reviews can be considered PIOs. However, the quality of evidence for these outcomes remains limited.

## Conclusions

Our study confirms that many outcomes reported in the SoF tables of recent Cochrane reviews can be considered PIOs. However, the quality of evidence for these outcomes remains limited.

## Supporting information

S1 Table Quality of available evidence for outcomes reported in the SoF tables for the main comparison in 290 recent Cochrane reviews: Comparison of all outcomes, to PIOs and surrogate outcomes. (DOCX)

S1 File Supporting data 1: Complete list of outcomes, classified and categorized. (XLSX)

S2 File Supporting data 2: All the study data. (XLSX)
