# Supplementary material for: Patient-important outcomes in systematic reviews: Poor quality of evidence
Source: PLoS One. 2018 Apr 5;13(4):e0195460. doi: 10.1371/journal.pone.0195460 (PMC5886560; doi:10.1371/journal.pone.0195460)
Supplement: S1 Table — (DOCX) [file pone.0195460.s001.docx]

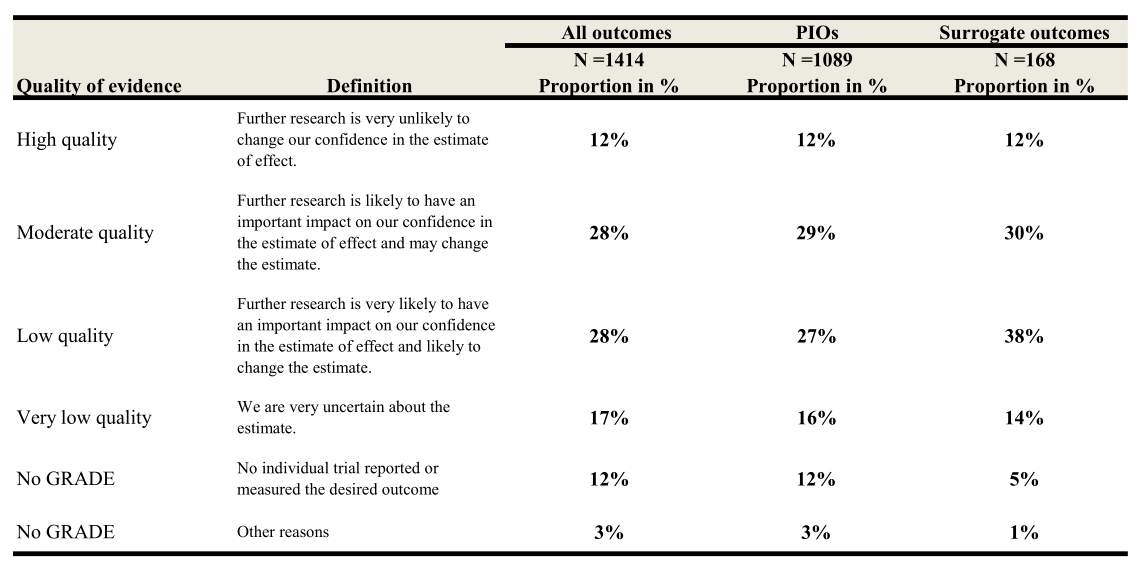
Supporting table 1. Quality of available evidence for outcomes reported in the SoF tables for the main comparison in 290 recent Cochrane reviews
